# Effect of Cellulose and Cellulose Nanocrystal Contents on the Biodegradation, under Composting Conditions, of Hierarchical PLA Biocomposites

**DOI:** 10.3390/polym13111855

**Published:** 2021-06-02

**Authors:** Luciano Miguel Galera Manzano, Miguel Ángel Ruz Cruz, Nora Magally Moo Tun, Alex Valadez González, José Herminsul Mina Hernandez

**Affiliations:** 1 Unidad de Materiales, Centro de Investigación Científica de Yucatán, A.C., Calle 43 #. No 130, Col. Chuburná de Hidalgo, Mérida 97205, Yucatán, Mexico; luciano.galera@cicy.mx (L.M.G.M.); miguel.ruz@cicy.mx (M.Á.R.C.); noram_22@hotmail.com (N.M.M.T.); 2 Grupo Materiales Compuestos, Universidad del Valle, Calle 13 No. 100-00, Cali 76001, Colombia; jose.mina@correounivalle.edu.co

**Keywords:** hierarchical biocomposites, factorial design, microfibrillated cellulose, cellulose nanocrystals, composting, biodegradation

## Abstract

In this work, the effect of microfibrillated cellulose (MFC) and cellulose nanocrystals (CNCs) on the biodegradation, under composting conditions, of hierarchical PLA biocomposites (HBCs) was studied using a full 2^2^ factorial experimental design. The HBCs were prepared by extrusion processing and were composted for 180 days. At certain time intervals, the specimens were removed from the compost for their chemical, thermal and morphological characterizations. An ANOVA analysis was carried out at different composting times to study MFC and CNCs’ effects on biodegradation. The specimen’s mass loss and molecular weight loss were selected as independent variables. The results show that the presence of MFC enhances the PLA biodegradation, while with CNCs it decreases. However, when both cellulosic fibers are present, a synergistic effect was evident—i.e., in the presence of the MFC, the inclusion of the CNCs accelerates the HBCs biodegradation. Analysis of the ANOVA results confirms the relevance of the synergistic role between both cellulosic fibers over the HBC biodegradation under composting conditions. The results also suggest that during the first 90 days of incubation, the hydrolytic PLA degradation prevails, whereas, beyond that, the enzymatic microbial biodegradation dominates. The SEM results show MFC’s presence enhances the surface biodeterioration to a greater extent than the CNCs and that their simultaneous presence enhances PLA biodegradation. The SEM results also indicate that the biodegradation process begins from hydrophilic cellulosic fibers and promotes PLA biodegradation.

## 1. Introduction

Two of the main disadvantages of synthetic plastics today are that they are produced from petrochemical compounds and that their waste remains in the environment for long periods. Increasing pressure on manufacturers by new environmental and waste management policies, consumer demand, and the escalating oil prices drive trends in composite technology away from traditional materials. The tendency is to use green composite materials to replace common plastics in consumer products to improve performance while reducing weight and cost [1,2].

Polylactic acid (PLA) is linear aliphatic polyester that meets ASTM biodegradability requirements and can fully biodegrade in soils and under composting conditions without any problem [3,4,5,6]. However, its brittleness, low heat distortion temperature, and low- impact resistance restrict its use in high-performance applications. To improve its performance under high demands while maintaining its biodegradability, various researchers have incorporated cellulose fibers [7,8,9,10,11] or cellulose nanofibers [12,13,14,15,16,17] to increment its HDT and improve its impact properties, among other mechanical and thermal properties.

As a result of these research efforts, PLA is used today in a wide variety of applications [18,19,20]. In order to increase the competitiveness of biodegradable composite materials in such a way that they can displace conventional non-biodegradable materials in very dynamic and demanding sectors such as packaging, construction, textiles, and the automotive industry, a new generation of hierarchical or multiscale biodegradable composite materials (HBC) containing micro- and nanosized mechanical reinforcements are currently being developed [21,22,23,24,25,26,27].

The biodegradability of PLA [28,29,30,31,32] and its composites containing microsized cellulose [33,34,35,36,37] or nanocellulose [38,39,40,41,42] are well documented. These studies have shown that PLA is capable of biodegradation under both aerobic and anaerobic conditions and that it is more recalcitrant to biodegradation than polycaprolactone (PCL) and polyhydroxybutyrate (PHB). Regarding the biodegradability of PLA, the results of these studies show, in general terms, that microsized cellulose increases the susceptibility of PLA to biodegrade [24,33,43,44]. On the other hand, regarding the nanosized cellulosic fibers, the results are contradictory; some studies show that it tends to increase the biodegradation of PLA [41,42,45], while others indicate that it decreases it [13,39].

In addition to this, and as far as we know, studies aimed at studying the biodegradation, under composting conditions, of hierarchical PLA composites have not yet been carried out. In other words, the role that the type of cellulosic fibers and their quantity plays in the biodegradability of PLA during composting is unknown. This knowledge will undoubtedly be of great use to subsequently carry out a correct disposal of these new materials at the end of their useful lives.

Based on the above, this work aims to study, using a Design of Experiments (DOE) approach, the effect of the type of cellulosic fibers (microsized and nanosized) and their contents on the biodegradation of hierarchical PLA composites (HBCs). A Factorial Experimental Design (FED) is a DOE class that allows us to study the effects of two or more factors and their interactions on a response of interest [46]. The use of a FED to understand the relevance and possible interaction of the factors under study was used to contribute to the understanding of the biodegradation of HBCs when composting. As shown in previous studies [9,47,48,49,50], a FED involves the study of the influence of multiple factors (independent variables) on an experimental response or responses (dependent variables) [46]. In this work, a full 2^2^ factorial design with a central point was chosen, since this kind of FED is a rotatable orthogonal design that allows us to inspect the possible presence of curvature in the experimental region studied. Two responses were chosen for the analysis: the drop in the molecular weight of PLA, representative of the chemical structure, and the weight loss of the sample, as an indicator of composite bioassimilation. The HBC composites were prepared by extrusion and composted for 180 days. At certain time intervals, the specimens were removed from the compost for their chemical, thermal and morphological characterizations and later an ANOVA analysis was performed using the percentage of mass loss of the samples and the percentage of molecular weight loss as dependent variables.

## 2. Materials

PLA was purchased from NatureWorks^®®^ 3251D, with an average molecular weight (Mn) of 47.4 kg/mol, a glass transition temperature (Tg) of 59 °C, and a melting point (Tm) of 170 °C. The microfibrillated cellulose (MFC) used was obtained from henequen fibers (*agave fourcroydes*) using a procedure detailed in [9] and was 500 ± 35 µm in length, on average, and 12 ± 2 µm in diameter, on average. The cellulose nanocrystals (CNCs) were purchased from The University of MAINE Process Development Center, having an average of 296 ± 10 nm in length and 21.4 ± 2 nm in diameter. Three-month-old mature food waste compost was used for the composting process.

## 3. Experimental Procedure

### 3.1. Statistical Experimental Design

A full 2^2^ factorial design with three central points was used to assess the effect of cellulose (MFC) and cellulose nanocrystal (CNC) contents on the biocomposites during the composting time. The response variables chosen were biocomposites weight loss and the PLA molecular weight loss. Analyses of variance (ANOVAs) with *p* = 0.05 were performed using the software Design-Expert 7.0 (Stat-Ease, Inc., Minneapolis, MN, USA). Pareto plots were used to visualize the effects of the factors or their interactions and the surface response and contour plots to analyze the effect of cellulose and CNC contents during the incubation time. The factor levels studied and experimental matrix obtained according to factorial design are shown in Table 1.

### 3.2. Composite Preparation

The hierarchical biocomposite materials (HBCs) were made using a BRABENDER mixing chamber PLE-330 plasticorder (C.W Brabender Instruments Inc., South Hackensack, NJ, USA) with three heating zones. The temperature profile used was 170 °C in the 3 zones, the speed of the blades was 50 rpm, and the mixing time was 20 min. Composites were molded by compression molding press in a Carver MH 4389-4021 semiautomatic press (Carver Inc., Wabash, IN, USA) equipped with heating plates and a forced water circulation cooling system. To shape the samples, stainless steel molds were used to generate square biocomposite plates with widths and thicknesses of 120 and 1 mm, respectively. The press temperature was 170 °C, and the pressure was 5500 lb. From the laminates, specimens of dimensions 18 × 5 × 1 mm were obtained. The specimens were dried at 50 °C under vacuum until a constant weight was reached, and then the specimens were stored in glass desiccators at a constant temperature of 25 ± 1 °C and humidity 25 ± 2%.

### 3.3. Biodegradation—Composting Setup

The HBC specimens were placed in an IM4000 rotary drum composter of 140 L capacity (Forest City Models and Patterns Ltd., Thorndale, ON, Canada). The main unit of the composter, i.e., the drum, is made of polypropylene 0.71 m in length and 0.91 m wide. The drum was rotated manually once every 24 h for proper mixing, and aerobic conditions were maintained by opening up both half side doors of the drum after rotation. Three-month-old mature food waste compost was used [51]. The C/N ratio used was 25:1, pH = 7, and the temperature was controlled at around 40–50 °C during the composting.

### 3.4. Analytical Characterization

#### 3.4.1. Scanning Electronic Microscopy (SEM)

The morphological evolution of the sample surfaces during composting was characterized using a scanning electron microscope JEOL SEM model LV5400 (JEOL, Mexico City, Mexico) operated at 20 kV. The samples were previously covered with gold.

#### 3.4.2. Weight Loss Monitoring

The weight loss (*w_L_*) of the hierarchical composite materials during composting was determined as follows: before the composting process, the samples were dried at 50 °C/24 h/with vacuum until constant weight (*w*_1_) was reached. Then, they were buried in the compost for 6 months. A group of samples was taken every 30 days. They were carefully cleaned with distilled water and vacuum-dried at 50 °C/24 h until they reached constant weight (*w*_2_). The percentage of weight loss was calculated with the following equation [11]:

(1) $$w_{L}=\left(\frac{w_{1}-w_{2}}{w_{1}}\right)\times 100$$

where:

*w*_1_ = is the initial dry sample weight;

*w*_2_ = is the dry weight of a sample after composting biodegradation.

#### 3.4.3. Gel Permeation Chromatography (GPC)

The molecular weight determination of the HBCs was carried out using a Gel Permeation Agilent Chromatograph 1100 series, (Agilent Technologies, Inc., Santa Clara, CA, USA). Samples were prepared for analysis by dissolving 5 mg in 5 mL of HPLC grade chloroform.

#### 3.4.4. Infrared Fourier Transformed Spectroscopy (FTIR)

Infrared analysis was performed using a Thermo Scientific Nicolet 8700 spectrophotometer (Thermo Electron Scientific Instruments LLCD, Madison, WI, USA). The samples were analyzed using the photo-acoustic mode.

#### 3.4.5. Differential Scanning Calorimetry (DSC)

Differential scanning calorimetry analysis was carried out on a Perkin Elmer DSC7 differential scanning calorimeter (Perkin Elmer, Inc., Waltham, MA, USA). The samples were analyzed using a heating ramp of 10 °C/min, in a range of 45–200 °C in an N_2_ atmosphere. The sample crystallinity content, *X_C_*, was computed according to Equation (2):

(2) $$X_{c}\left(\%\right)=\frac{\Delta H_{m}-{\Delta H}_{cc}}{\Delta H_{m}^{0}w_{PLA}}$$

where: Δ*H_m_* is the composite’s fusion enthalpy, Δ*H*^0^*_m_* is the 100% crystalline *PLA*’s fusion enthalpy taken as 93 J/gr [42], ΔH*_CC_* is the cold crystallization enthalpy, and *W_PLA_* is the weight fraction of the *PLA* phase in the composite.

## 4. Results

### 4.1. Morphological Analysis

**Figure 1:**
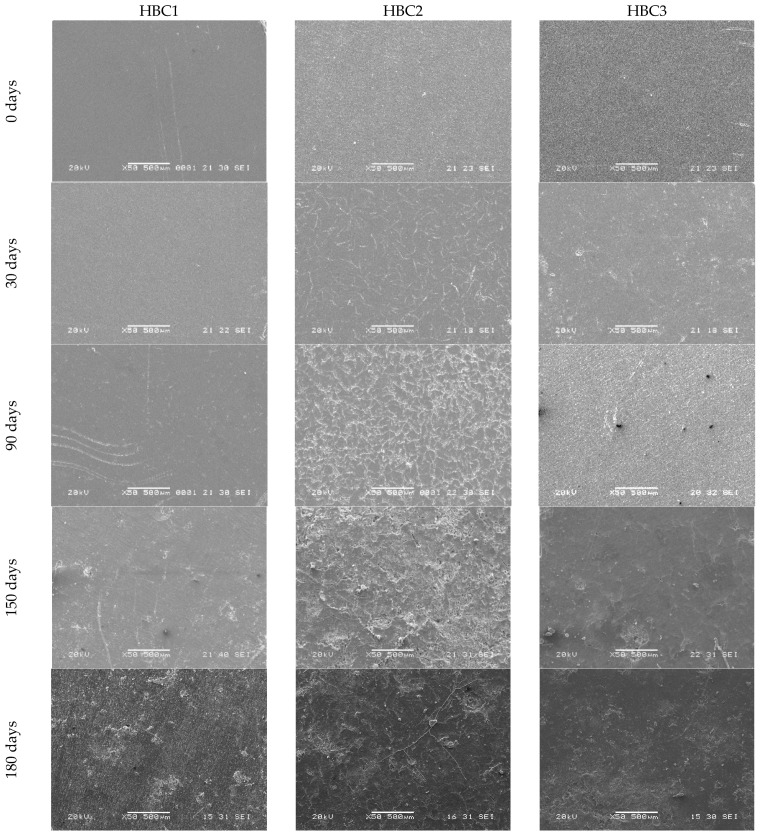
HBC1, HBC2 and HBC3 SEM micrographs at different composting days (0, 30, 90, 150, and 180 days).

**Figure 2:**
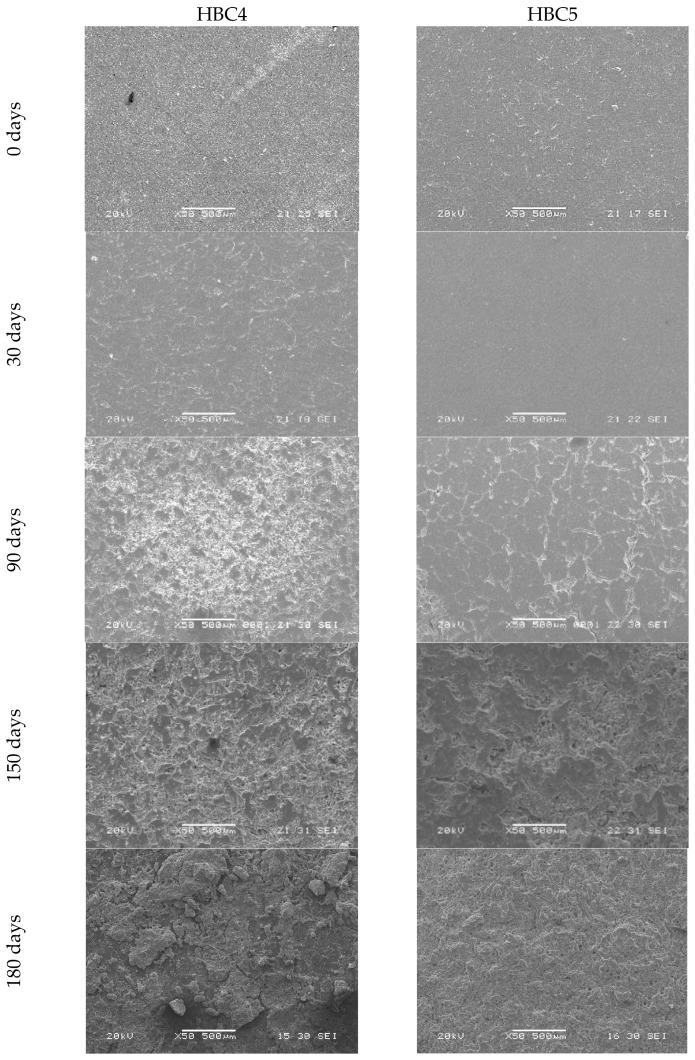
HBC4 and HBC5 SEM micrographs at different composting days (0, 30, 90, 150, and 180 days).

The change in the surface morphological characteristics of the HBCs studied due to being subjected to different incubation days in a rotary composter can be seen in Figure 1 and Figure 2. While all the samples have essentially smooth surfaces before being composted (0 days), after 30 days of incubation, evident signs of surface deterioration can be seen mainly in HBC2 (Figure 1) and HBC4 (Figure 2), samples that include MFC and MFC/CNCs in their compositions, respectively. Although HBC5 also contains MFC and NCC, the changes are very slight. This may be due to the fact that the contents of said cellulosic fibers are half with respect to HBC4. On the other hand, neither HBC1 nor HBC3 show appreciable surface damage, although HBC3 contains 5% NCC. This apparent lack of surface damage during the first 30 days of incubation could be attributed to the hydrophobic nature of PLA [3,5]. These findings suggest the addition of the cellulosic fibers decreases the PLA hydrophobicity due to cellulosic fibers’ hydrophilic natures [13,14,42], enhancing their biodegradability.

When examining the samples with 90 days of composting, one can see that the deterioration of the samples is much more evident in the samples that contain cellulosic fibers (HBC2, HBC3, HBC4, and HBC5) compared to the one that does not have any (HBC1). Likewise, it can be seen that the higher the fiber content, the greater the evidence of surface damage, e.g., HBC4 > HBC2 > HBC5 > HBC3. This behavior agrees with literature reports that the presence of cellulosic fibers promotes PLA biodegradation [24,33,38,45]. At 150 days of incubation, all the HBCs show evidence of surface deterioration, including HBC1. Furthermore, roughness, the presence of small holes or cracks, and even signs of erosion are evident in HBC2, HBC4 and HBC5, with these changes being much more noticeable in HBC4 and HBC2 than in HBC5. The micrographs of the samples incubated for 180 days continue to show the trend described above for 150 days; the samples show clear signs of biodeterioration in the order HBC4 > HBC2 > HBC5 >> HBC3 > HBC1.

**Figure 3:**
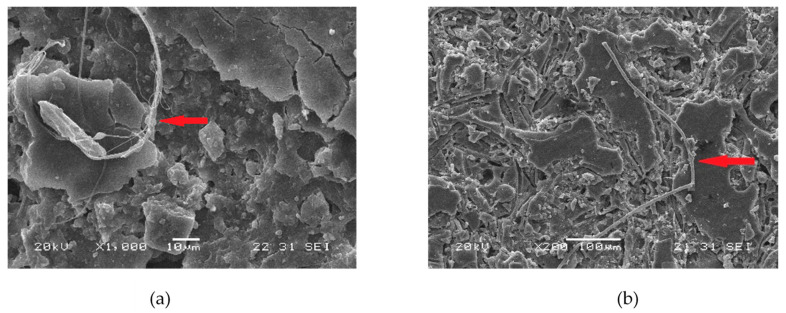
Micrographs of HBC2 (**a**) and HBC4 (**b**) surfaces composted for 150 days. The red arrow shows the presence of a microbial hypha formed during composting.

It should be noted that the HBC4 samples show the greatest surface erosion, suggesting that they are the ones that underwent the greatest biodegradation. These findings suggest that although the addition of cellulosic fibers separately (MFC or CNC) promotes the biodegradation of PLA, their simultaneous presence enhances biodeterioration. In Figure 3a,b, SEM micrographs of HBC2 and HBC4 with 150 days of composting are shown, respectively. In both micrographs, the presence of microorganisms was observed on both the surface of the sample and surrounding the cellulosic fibers. These findings indicate that the biodegradation process began from hydrophilic fillers and then promotes PLA biodegradation [14,33]. In Figure S1 (Supplementary Data), the fracture surface SEM micrographs of HBC2 and HBC4 before composting are shown, where it can be seen that the NCC tends to adhere to the MFC surface S1 (d) and S1 (e). Additionally, in Figure S2, the degree of deterioration of the HBCs can be observed at three different resolutions, ×100, ×1000 and ×2000 for HBC1 (a–c), HBC2 (d–f), HBC3 (g–i) and HBC4 (j–l) after 180 days of composting.

### 4.2. Weight Loss

**Figure 4:**
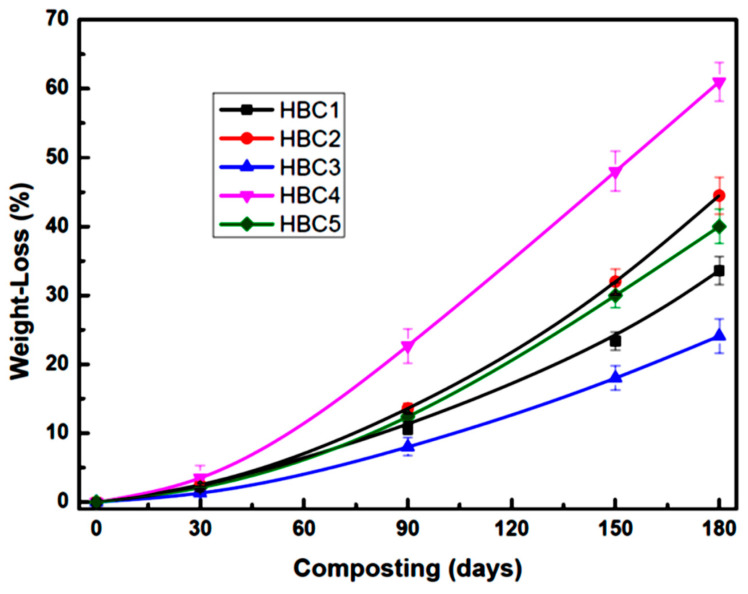
HBCs weight loss changes during composting.

The HBC weight loss curves for different incubation times are shown in Figure 4, where it can be seen that, as expected, the weight loss rises as the incubation time increases, although not at the same rate for all biocomposites. During the first 30 days, all HBCs show similar weight loss, and it is up to 90 days of incubation that differences can be perceived between them. It is even noticeable that weight loss increases significantly after 90 days of incubation at different rates as the incubation time increases. It is interesting to note that cellulose microfibers (HBC2) addition results in a much more significant weight loss than the PLA matrix (HBC1), while the inclusion of cellulose nanocrystals reduces the biocomposite weight loss.

This difference in the behavior of MFC and CNC in the biodegradation of PLA can be attributed to the fact that, as cellulose fibers’ contents are much higher, they give the composite material a greater hydrophilic character, which facilitates the attack of microorganisms [52,53]. On the other hand, the low content of CNCs does not modify the hydrophobic character of PLA appreciably. Likewise, CNC’s nucleating power increases the crystalline phase of PLA, which is difficult for the microorganisms to attack [13,39]. As can be seen in Figure 4, the simultaneous presence of MFC and CNC increases the weight loss of the biocomposites after 90 days of incubation and practically doubles the weight loss of PLA after 180 days of composting. Considering the weight loss as a measure of the bioassimilation of the material by the microorganisms present in the compost and the weight loss of HBC1 after 180 days as a reference (100%), we see that the presence of MFC (HBC2) increases biodegradation by 46%, the inclusion of CNC (HBC3) decreases it by 21%, while the simultaneous presence of MFC and CNC (HBC4 and HBC5) increases biodegradation by 60% and 21%, respectively. This behavior suggests that there is a synergistic effect between MFC and CNC.

### 4.3. Molecular Weight Analysis

**Figure 5:**
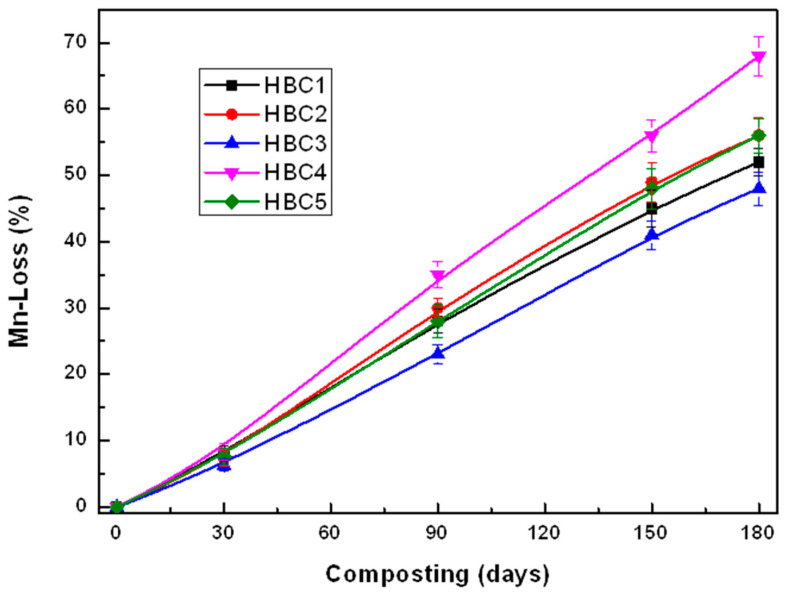
HBC molecular weight loss changes during composting.

Figure 5 shows the loss in average molecular weight (Mn) of HBCs with composting time. As can be seen, the drop in Mn is similar for all biocomposites during the first 30 days, and, thereafter, differences in Mn loss are observed between them. Furthermore, during all the composting, the drop in Mn is greater for all compounds, except HBC3, compared to HBC1, suggesting that the inclusion of CNCs (HBC3) inhibits the abiotic and biotic degradation of PLA. This loss in molecular weight is due to the combined effect of the abiotic (hydrolysis) and biotic (biodegradation) factors that affect the PLA matrix. Hydrolysis induces the cleavage of the chemical bond in the main chain by the reaction with water, initiated by protonation, followed by the addition of water and ester bond cleavage [13]. The degradation usually begins with the hydrolysis of the PLA chains induced by the diffusion of water within the materials.

The reduction in the molecular weight of PLA caused by the non-enzymatic random cleavages of the ester groups leads to the formation of oligomers and lactic acid. When the molecular weight reaches about 10,000–20,000 g.mol^−1^, microorganisms such as fungi and bacteria can metabolize macromolecules by converting them to carbon dioxide, water and humus; this step occurs on the surface of the material. The biodegradation in compost of PLA composites reinforced with microcrystalline cellulose was described as a slower disintegration rate and was observed for the composites and attributed to a higher resistance to water uptake and diffusion through the composites compared to pure PLA [38,54].

It should be noted that the inclusion of the CNCs alone delays the Mn loss [39], while the presence of MFC promotes it [43]. Likewise, when both cellulosic materials are present, the drop in molecular weight practically doubles the weight loss with respect to PLA at 180 days of composting. Considering the Mn loss of HBC1 after 180 days of incubation as a reference (100%), we see that the presence of MFC (HBC2) increases the Mn loss by 8%, while the inclusion of CNC (HBC3) decreases it by 21%. On the other hand, the simultaneous presence of MFC and CNC (HBC4 and HBC5) increases the Mn loss by 31% and 6%, respectively. These findings suggest that there is a synergistic effect between MFC and CNC during the PLA biodegradation under composting conditions.

### 4.4. Infrared Spectroscopy (FTIR) Analysis

**Figure 6:**
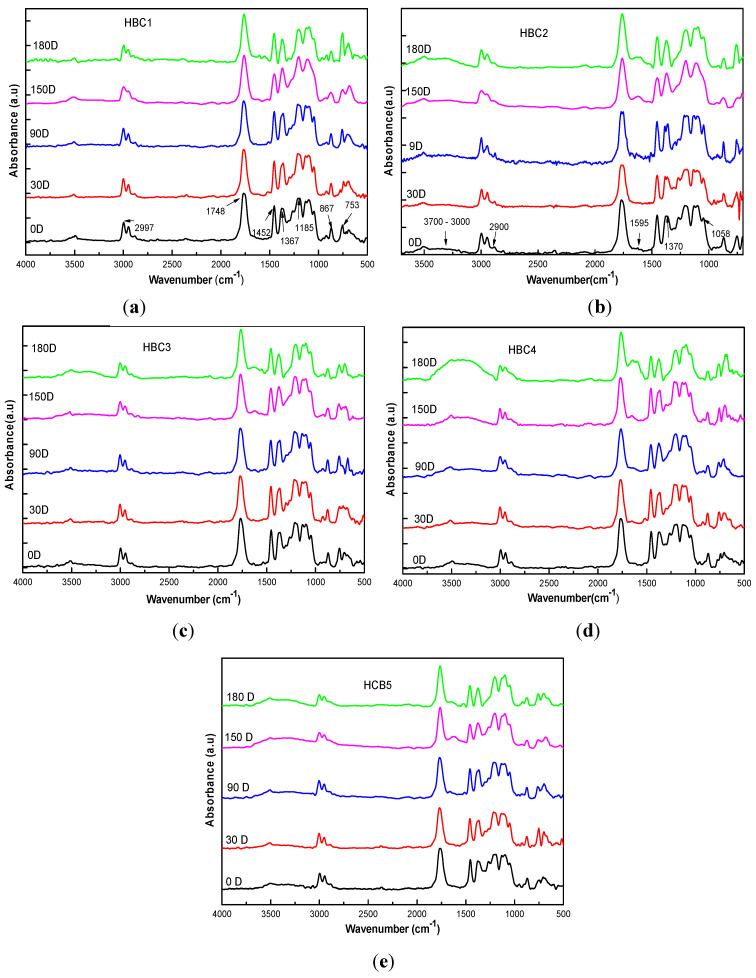
HBC infrared spectra for different composting days in samples: (**a**) HBC1; (**b**) HBC2; (**c**) HBC3; (**d**) HBC4; (**e**) HBC5.

The FTIR spectra of the HBCs studied at different incubation times are presented in Figure 6. Those corresponding to HBC1 are shown in Figure 6a, where the characteristic bands of PLA can be observed, located around 2997, 2940, 1760, 1457, 1045, 870, and 760 cm^−1^ in the uncomposted sample (0D). Likewise, it can be seen that there is a great similarity between the HBC1 spectra for all the different incubation times, which suggests that the by-products of PLA degradation were solubilized in water or were bioassimilated [55].

The infrared spectra of the HBC2 biocomposites as a function of the incubation time are presented in Figure 6b, where, in addition to the PLA peaks, the characteristic absorption bands of cellulose at 2890, 1370, 1160, 1110, and 900 cm^−1^ can be seen in the uncomposted material (0D) [56]. During the first 90 days of composting, there are no substantial changes in the spectra; however, at 150 and 180 days, the presence of new absorption bands can be observed at 3500, 1620, 1540 cm^−1^, which can be attributed to the presence of microorganisms on the surface of the samples [57]. It can also be observed that the intensity of the characteristic bands of cellulose at 2890 cm^−1^ increases concerning those of PLA, indicating a more significant presence of cellulose on the surface of HBC2.

Regarding HBC3, the characteristic bands of the CNCs, which are essentially the same as those of microfibrillated cellulose, are observed in the spectrum corresponding to 0D shown in Figure 6c. Likewise, it can be observed that the spectra corresponding to the first 150 days of incubation are similar, and it is the samples that were incubated for 180 days that the presence of new absorption bands at 3500, 3300, 1620, 1540 cm^−1^ is evident. However, there is not observed an increase in the intensity of the CNC bands that could suggest a greater presence of them on the surface of HBC3, as occurs in HBC2. This may be because the low content of CNCs in the composite makes it difficult to identify using FTIR.

As far as HBC4 is concerned, in Figure 6d, it can be seen that the spectrum corresponding to 0D is very similar to those described above for uncomposted HBC2 and HBC3. This is expected since both types of cellulosic fibers, MFC and CNC, are found in HBC4. However, the behavior observed in the HBC4 spectra with the incubation time is more similar to those described for HBC2 than for HBC3 but is much more intense. The intensity of the bands at 3500, 3300, 1620, and 1540 cm^−1^ are much higher, indicating that there is a greater degree of biodegradation in HBC4 compared to HBC2 and HB3, which contain only MFC or CNC, respectively [57,58]. This suggests that there may be some synergy between cellulosic fibers during the biodegradation process.

Finally, in Figure 6e, it can be seen that HBC5 shows an intermediate behavior between HBC4 and HBC1. This could be attributed to the low contents of MFC and NCCs.

### 4.5. Differential Scanning Calorimetry (DSC) Analysis

**Figure 7:**
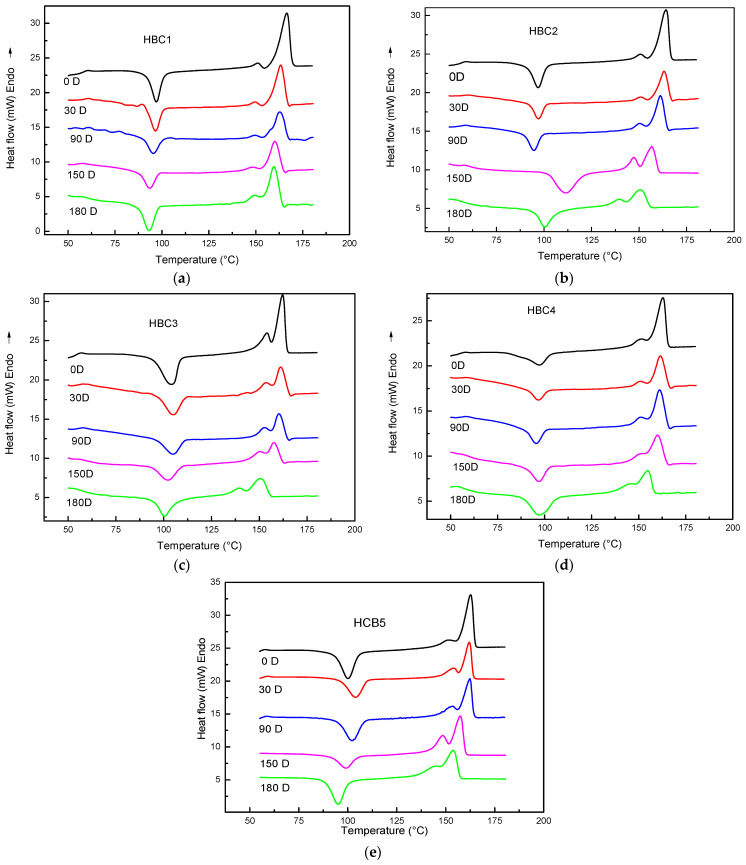
HBC thermograms for different composting days in samples: (**a**) HBC1; (**b**) HBC2; (**c**) HBC3; (**d**) HBC4; (**e**) HBC5.

The HBCs first heating thermograms for different composting days are shown in Figure 7, where it can be seen, as expected, that the characteristic thermogram of a semicrystalline PLA are clearly distinguished in all the HBCs without composting (0D), e.g., the Tg peak around 60 °C, the cold crystallization temperature (Tcc) at 100 °C and the melting temperature (Tm) at 160 °C. Additionally, it can be observed that as the incubation duration increases, these transition peaks suffer specific positional changes that are summarized in Table 2, along with the sample crystallinity content, computed with Equation (2).

An analysis of the data in Table 2 shows that in the case of HBC1, which only contains PLA, it has a glass transition temperature (Tg) of 60 °C at 0D. It remains constant the first 90 days of incubation and then drops to 58 °C with 180D. A similar trend can be observed for the cold crystallization temperature (Tcc), first melting temperature (Tm_1_), and the second one (Tm_2_), e.g., they remain constant the first 90 days of incubation and then drop.

This behavior indicates that a loss of 25–30% in molecular weight is not reflected in Tg, Tcc, and Tm changes, while a decrease of 50% or more in Mn causes Tg, Tcc, and Tm to shift to lower temperatures. Similar behavior has been reported in [59,60]. The sample crystallinity content Xc (%) after processing, in the case of 0D, and just as it is removed from the compost bin in the different incubation days, is also shown in Table 2. It can be seen that Xc (%) shows a different behavior; it increases during the first 90 days and then gradually drops as the incubation time increases. This behavior can be attributed to the fact that during the first 90 days of incubation, PLA undergoes a hydrolytic degradation that mainly affects the amorphous fraction capable of crystallizing. After 90 days of incubation, PLA undergoes hydrolytic degradation and enzymatic degradation due to microorganisms that affect both the amorphous and crystalline phases [3,30]. Likewise, it has been reported that in the final stages of biodegradation, PLA loses the ability to crystallize due to the presence of by-products of biodegradation [30].

In the case of HBC2, the Tg at 0D is similar to that of HBC1 and shows a similar trend with the incubation time. During the first 90 days, the Tcc of HBC2 is similar to HBC1; however, after that, the Tcc of HBC2 is higher than HBC1. The Tm_1_ and Tm_2_ of HBC2 differ from that of HBC1 after 150 days of incubation and they are lower. On the other hand, the Xc (%) contents for HBC2 are higher than HBC1 at 0D, increasing during the first 30 incubation days, and decreasing steadily afterwards. These differences in behavior between HBC1 and HBC2 can be attributed to the presence of MFCs, which promote, on the one hand, more significant crystallization of PLA at short biodegradation times and a higher biodegradation rate due to its greater hydrophilicity on the other. During hydrolytic degradation, they promote the growth of PLA crystals, whereas the presence of microorganisms accelerates the biodegradation [24,33,43,44,45]. For HBC3, the Tg values are slightly lower than HBC1 and HBC2, whereas their behavior is related to the incubation time being similar to these two. A similar behavior can be observed regarding Tcc, i.e., the starting value is higher than HBC1 and HBC2, while its behavior with the incubation time is similar to both of them, i.e., it remains constant for the first 90 days and then decreases. The crystallinity content Xc (%) at 0D is greater than HBC1 but less than HBC2. Its behavior concerning incubation time is more similar to HBC2 than to HBC1—that is, it increases during the first 30 days of incubation and gradually decreases.

Due to the erosion of the composite material, the high hydrophilicity of CNCs promotes the biodegradation of PLA. These results confirm that CNCs have a great capacity to promote crystallization at short times, whereas at long times they enhance biocomposite biodegradation [36,41,42].

With respect to HBC4, although the value of Tg at 0D is similar to the other three HBCs, its behavior with the incubation time is different. Tg steadily decreases as time increases. The Tg value at 180 days of composting is the lowest of the four studied biocomposites. The value of Tcc is similar to those of HBC1 and HBC2 but less than HBC3, and its evolution with the incubation time is similar to the other ones. Regarding Tm_1_ and Tm_2_, the observed behavior is much more similar to HBC1 than to HBC2 and HBC3. Regarding Xc (%), at 0D, the value is higher than all other materials, and its change concerning the incubation time is similar to HBC2 and HBC3—that is, it increases in the first thirty days and subsequently decreases. However, it is important to note that the drop is a bit more pronounced in this case. The crystallinities of the HBC4 samples, after 150 days of composting, are the lowest considering all the materials studied. Finally, HBC5 samples show an intermediate behavior between HBC4 and HBC1 during the composting, similar to the one pointed out in the FTIR results.

These results suggest two-stage degradation for PLA, a hydrolytic degradation at a duration of less than 90 days for incubation and an enzymatic degradation induced by the proliferation of microorganisms at a duration of greater than 90 days for composting. There are also indications of a possible synergistic effect between MFC and NCC, especially in the second stage of PLA degradation.

### 4.6. Statistical Experimental Design

**Figure 8:**
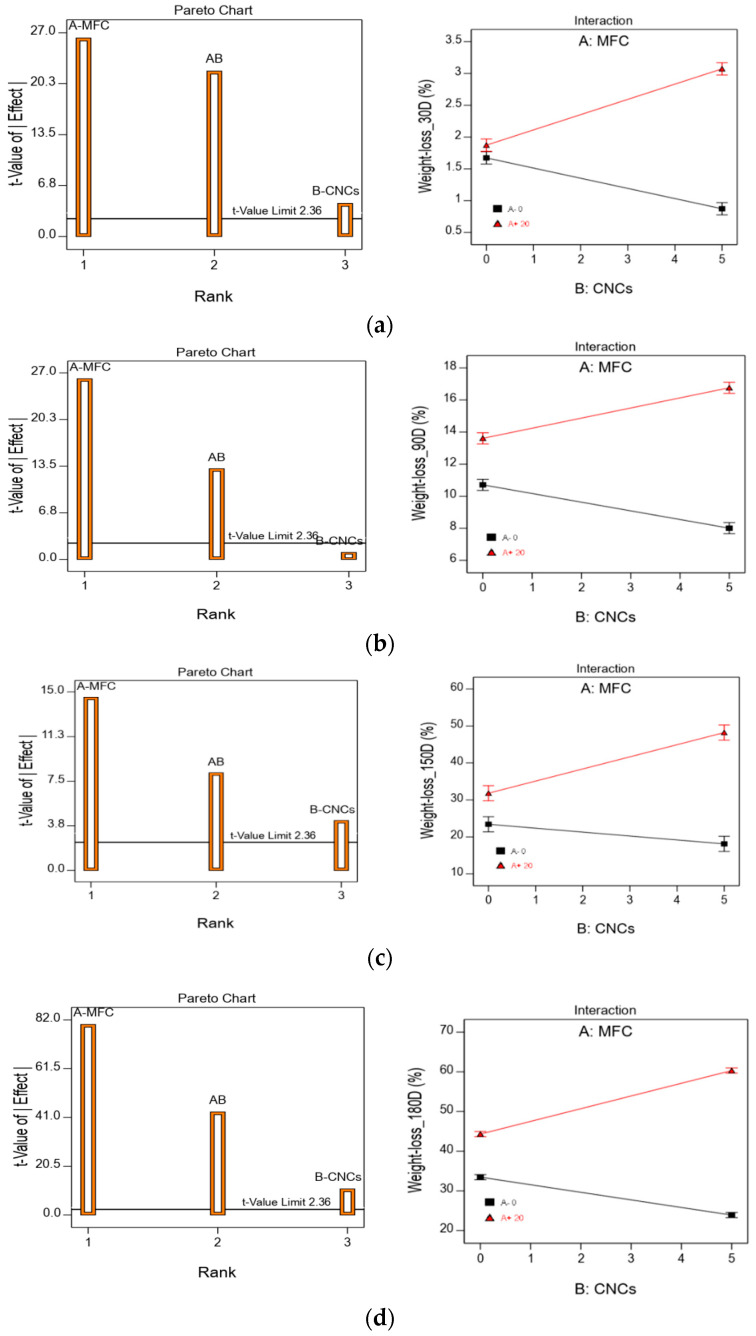
Pareto and interaction graphs for HBCs weight loss for (**a**) 30 D; (**b**) 90 D; (**c**) 150 D; and (**d**) 180 D incubation days.

With the aim to study the role that both MFC and CNC have in HBC biodegradation under composting conditions, an analysis of variance (ANOVA) was carried out at 30, 90, 150, and 180 days of incubation. The HBC weight loss and the Mn loss were selected as the dependent variables. The results of the ANOVA analysis for the HBC weight loss for the different incubation times can be seen in the Pareto graphs shown in Figure 8. As can be seen, for any composting time, the MFC contents is the variable that has the most influence on weight loss, followed by the MFC–CNC interaction and at last by the CNC contents. The relevance of the MFC–CNC interaction can be observed in the interaction graphs presented in Figure 8, where a dramatic change in the behavior of the CNC contents during the composting process can be seen. In absence of MFC (black line), as the CNC content increases, the HBCs weight loss decreases, i.e., the presence of CNCs only delays the composites biodegradation, whereas, in the presence of the MFC (red line), the behavior is the opposite—the composites weight loss increases with CNC content. Additionally, it can be noted that the slope of the black lines drops with the incubation times, suggesting the CNC’s delaying effect diminishes over long composting times.

**Figure 9:**
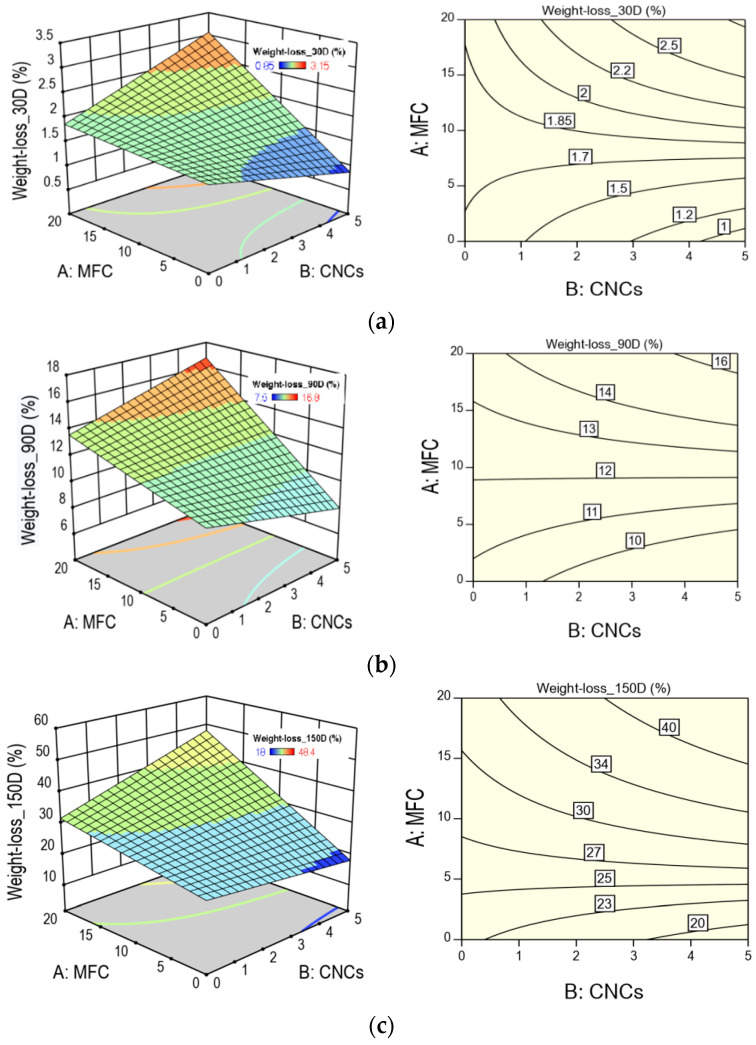

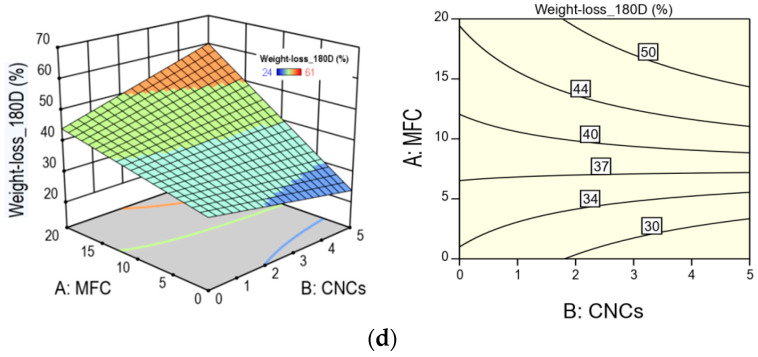
Surface response and contour plots for weight loss for (**a**) 30 D; (**b**) 90 D; (**c**) 150 D; and (**d**) 180 D incubation days.

Figure 9 shows the response surfaces and contour graphs for weight loss at different composting times. The characteristic warping can be seen in all the response surface graphs because the MFC–CNC interaction was statistically significant, while in the case of the contour graphs, it can be seen that the contour curves present curvature. In the absence of interaction, the contour lines would be straight lines. Likewise, the areas of greatest (red) and least (blue) biodegradation can be distinguished. It can also be observed that the effect of the incorporation of the CNCs in the HBCs is greater during the first 90 days of composting, while at extended times (greater than 90 days), its effect decreases. For example, 30 days after composting, the effect of incorporating 5% CNCs increases weight loss by 68% in the presence of 20% MFC and decreases biodegradation by 48% in the absence of MFC. At 180 days of composting, the increase is 40% and the decrease is of the order of 30% with and without MFC in the composite material.

**Figure 10:**
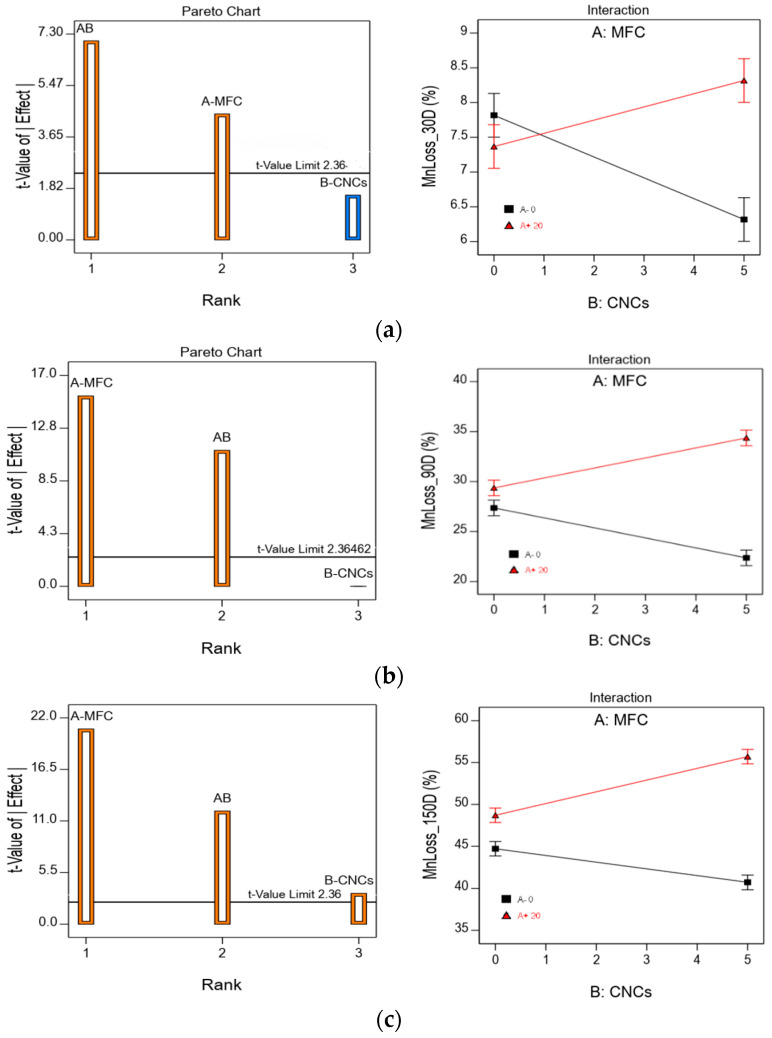

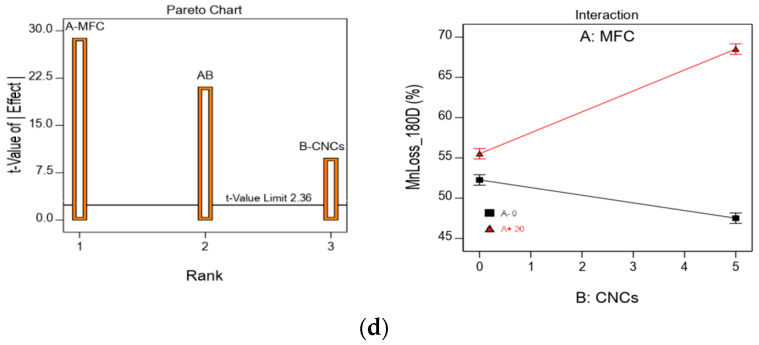
Pareto and interaction graphs for HBC Mn loss for (**a**) 30 D; (**b**) 90 D; (**c**) 150 D; and (**d**) 180 D incubation days.

Figure 10 shows the ANOVA results for the loss of molecular weight (Mn). In this figure, it can be seen that the trends in terms of the role played by both independent variables, MFC and CNC, in the drop in molecular weight are very similar to those described for weight loss—that is, there is a significant similarity in the way that the presence of both MFC and CNC affects biodeterioration and loss of molecular weight during composting.

**Figure 11:**
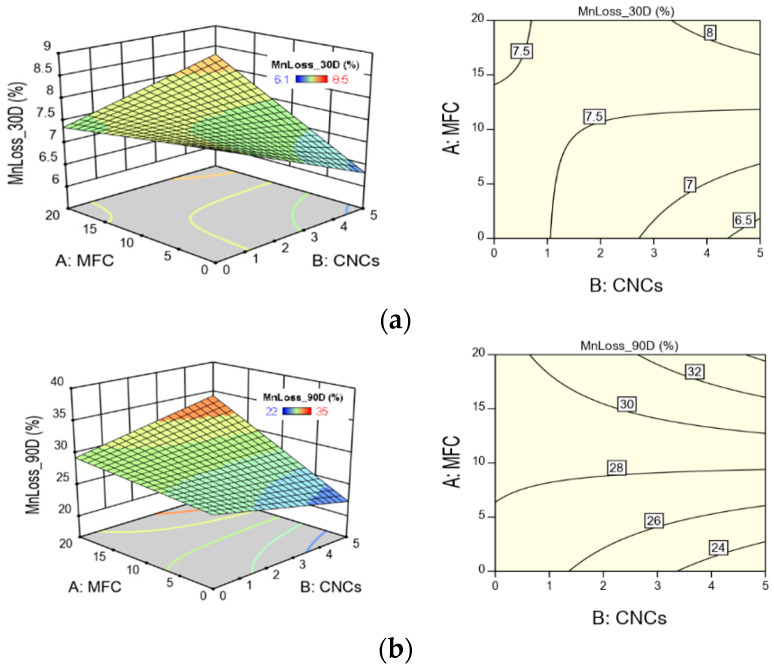

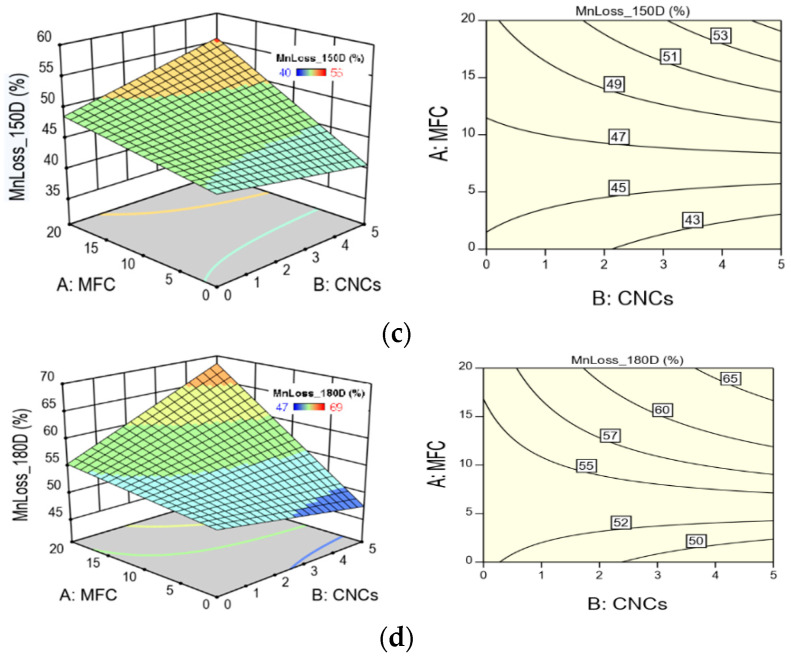
Surface response and contour plots for HBCs Mn loss for (**a**) 30 D; (**b**) 90 D; (**c**) 150 D; (**d**) 180 D incubation days.

Figure 11 shows the response surfaces and contour graphs for Mn loss at different composting times. The response surface graphs show that the characteristic twist due to the MFC–CNC interaction is present, whereas, in the case of the contour graphs, it can be seen how the contour curves present curvature. In the case of the 30D incubation days, the interaction is so strong that the contour lines resemble a hyperbolic plot. Likewise, the areas of greatest (red) and least (blue) biodegradation can be distinguished. It can also be observed that the effect of the incorporation of the CNCs in the HBCs is greater during the first 90 days of composting, while, at extended times (greater than 90 days), its effect decreases without MFC; in the presence of MFC, it does not depend on the composting time, i.e., it is almost constant. For example, after 90 days of composting, the effect of incorporating 5% CNCs increases the Mn loss by 20%, with MFC, and decreases it by 20% in the absence of MFC. At 180 days of composting, the increase is 24% and the decrease is of the order of 10% with and without MFC in the composite material.

## 5. Conclusions

The effect of the incorporation of microsized cellulose (MFC) and cellulose nanocrystals (CNCs) during the composting of hierarchical PLA biocomposites (HBCs) was studied in this work using a factorial experimental design.

The statistical analysis shows that MFC contents and the MFC–NCC interaction were statistically significant during all the composting processes for weight loss and Mn loss. The CNC contents show to be significant only during the last stage of composting.

Regarding the weight loss results, it was found that after 180 days of incubation, the presence of MFC increases the PLA biodegradation by 46%, while the CNCs decrease it by 21%. However, their simultaneous presence increases PLA biodegradation by 60%. Considering the Mn loss results, it was found that MFCs increase the PLA molecular weight drop by 8%, CNCs decrease it by 10%, and the simultaneous presence of MFCs and CNCs increases the Mn loss by 31%. This behavior suggests that the MFC–NCC interaction is a synergistic one.

The SEM results show that MFC’s presence enhances the surface biodeterioration over longer times when compared with the CNCs and that the HBCs containing both cellulosic fibers show the greatest surface biodeterioration, suggesting that their simultaneous presence enhances the PLA biodegradation. The SEM results also indicate that the biodegradation process begins from hydrophilic cellulosic fibers and promotes PLA biodegradation.

The FTIR and DSC results suggest two-stage degradation for PLA, a hydrolytic degradation during the first 90 days of incubation, and enzymatic degradation, induced by the proliferation of microorganisms, at times greater than 90 days. In addition, indications of a possible synergistic effect between MFC and NCC during the composting process, especially in the second stage of PLA degradation when the enzymatic microbial biodegradation dominates, were also found.

## Figures and Tables

**Table 1:** Factor levels and experimental design matrix.

	Levels	Factors	
		MFC (wt.%)	CNC (wt.%)
	−1 (lower)	0	0
	1 (higher)	20	5
Experimental matrix design			
	PLA (wt.%)	MFC (wt.%)	CNC (wt.%)
HBC1	100	0	0
HBC2	80	20	0
HBC3	95	0	5
HBC4	75	20	5
HBC5 (central point)	87.5	10	2.5

**Table 2:** Tg, Tcc, Tm_1_ and Tm_2_ and Xc of HBCs with 0, 30, 60, 90, 120 and 150 days of composting.

HBC1	0 Days	30 Days	90 Days	150 Days	180 Days
Tg (°C)	60	60	60	59	58
Tcc (°C)	97	97	95	94	93
Tm_1_ (°C)	151	150	150	149	149
Tm_2_ (°C)	166	163	162	160	159
Xc (%)	4	38	41	35	32
HBC2	0 days	30 days	90 days	150 days	180 days
Tg (°C)	59	59	59	58	58
Tcc (°C)	97	96	95	110	99
Tm_1_ (°C)	151	151	151	147	140
Tm_2_ (°C)	165	163	161	156	151
Xc (%)	9	43	32	27	21
HBC3	0 days	30 days	90 days	150 days	180 days
Tg (°C)	58	59	57	58	57
Tcc (°C)	102	104	104	101	100
Tm_1_ (°C)	154	154	153	151	140
Tm_2_ (°C)	162	161	160	158	151
Xc (%)	7	42	37	23	24
HBC4	0 days	30 days	90 days	150 days	180 days
Tg (°C)	59	58	57	56	55
Tcc (°C)	97	96	95	96	95
Tm_1_ (°C)	151	150	150	150	145
Tm_2_ (°C)	162	162	161	160	155
Xc (%)	12	45	31	22	11
HBC5	0 days	30 days	90 days	150 days	180 days
Tg (°C)	58	58	57	56	55
Tcc (°C)	98	96	95	96	96
Tm_1_ (°C)	152	151	151	151	141
Tm_2_ (°C)	161	161	160	159	152
Xc (%)	8	40	32	25	16

## Data Availability

The raw/processed data required to reproduce these findings cannot be shared at this time as the data also forms part of an ongoing study.

## Supplementary Materials

The following are available online at https://www.mdpi.com/article/10.3390/polym13111855/s1, Figure S1: Fracture surface Sem micrographs of HBC2 (a–b) and HBC4 (c–e) before composting; Figure S2: Sem micrographs at different resolutions for HBC1 (a–c); HBC2 (d–f); HBC3 (g–i) and HBC4 (j–l) after 180 composting days.
