# Supplementary material for: Effect of Cellulose and Cellulose Nanocrystal Contents on the Biodegradation, under Composting Conditions, of Hierarchical PLA Biocomposites
Source: Polymers (Basel). 2021 Jun 2;13(11):1855. doi: 10.3390/polym13111855 (PMC8199790; doi:10.3390/polym13111855)
Supplement: Supplementary file 1 [file polymers-13-01855-s001.zip › polymers-1218622-supplementary.pdf]

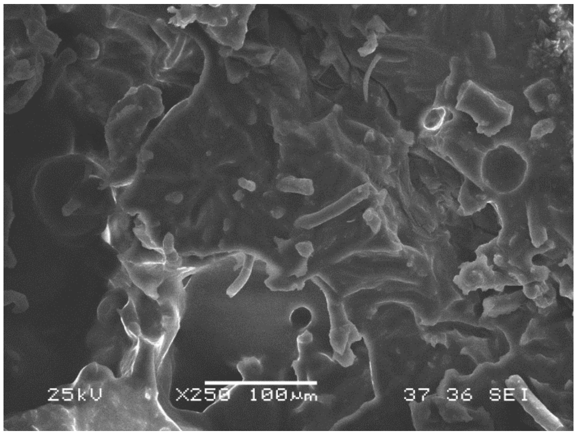

(a)

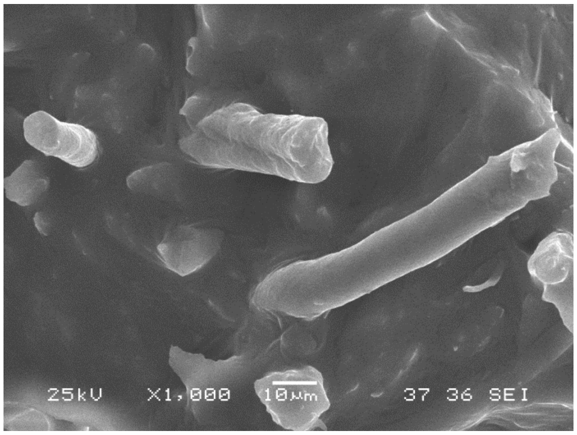

(b)

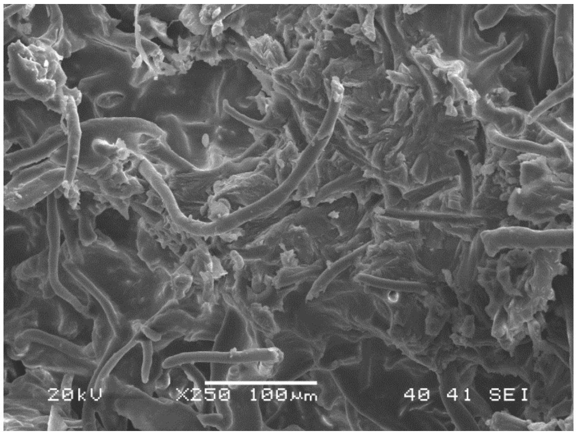

(c)

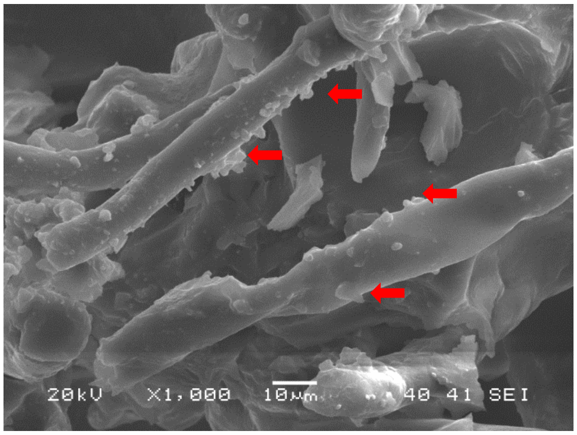

(d)

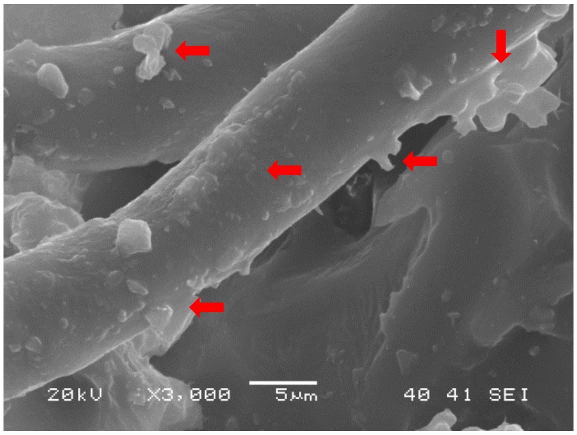

(e)

Figure S1. Fracture surface Sem micrographs of HBC2 (a-b) and HBC4 (c-e) before composting

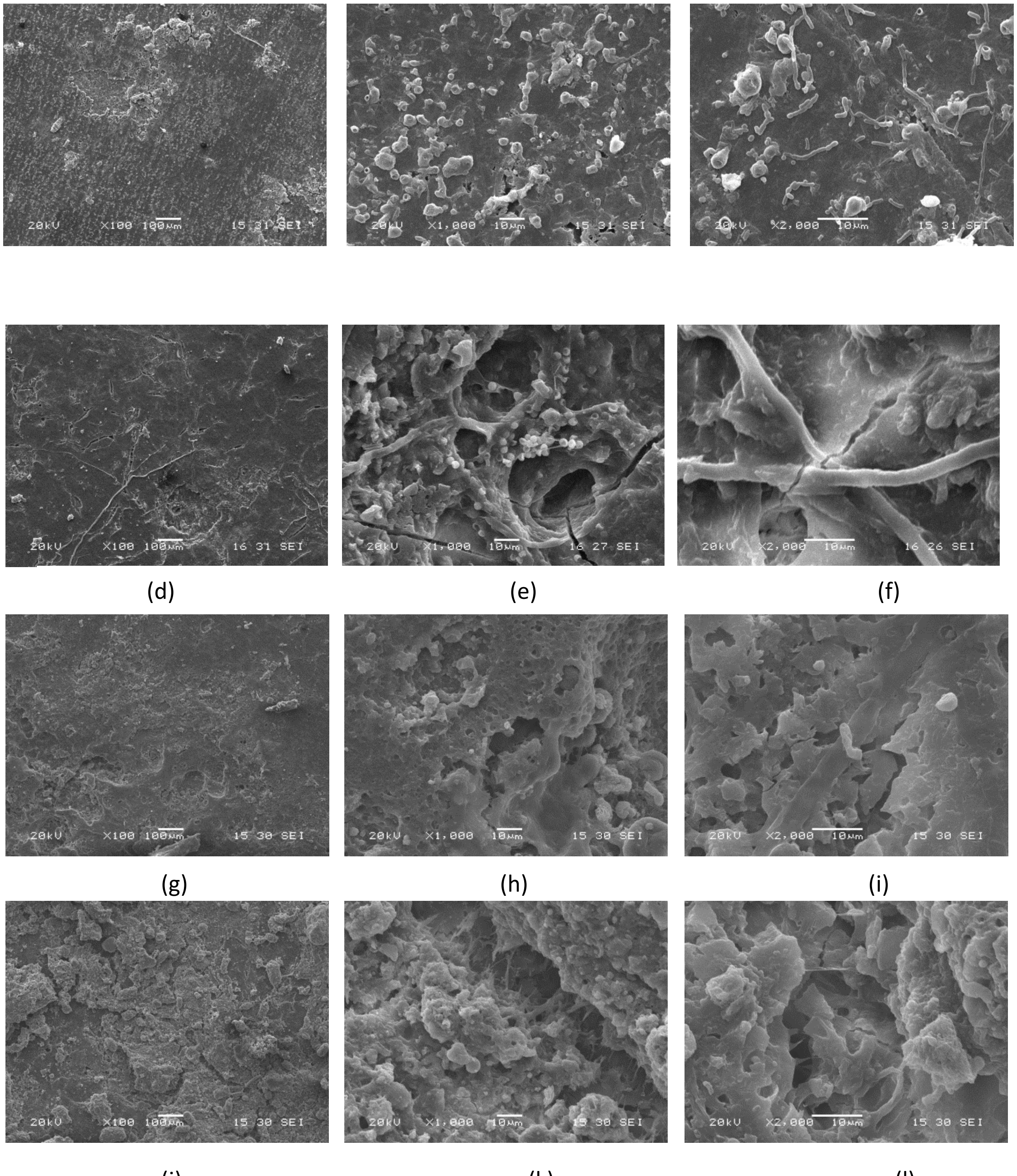

Figura S2. Sem micrographs at different resolutions for HBC1 (a-c); HBC2 (d-f); HBC3 (g-i) and HBC4 (j-l) after 180 composting days.
